# Association of cerebrospinal fluid NPY with peripheral ApoA: a moderation effect of BMI

**DOI:** 10.1186/s12986-024-00828-6

**Published:** 2024-07-25

**Authors:** Danyang Zhao, Xiaoli Han, Qingshuang Mu, Yan Wu, Ligang Shan, Lidong Su, Wenyan Wang, Pengxiang Wang, Yimin Kang, Fan Wang

**Affiliations:** 1 Medical Neurobiology Lab, Inner Mongolia Medical University, Huhhot, 010110 China; 2 Clinical Nutrition Department, Friendship hospital of Urumqi in Xinjiang, Urumqi, 830049 China; 3 Xinjiang Key Laboratory of Neurological Disorder Research, the Second Affiliated Hospital of Xinjiang Medical University, Urumqi, 830063 China; 4 Beijing Hui-Long-Guan Hospital, Peking University, Beijing, 100096 China; 5 Department of Anesthesiology, the Second Affiliated Hospital of Xiamen Medical College, Xiamen, 361021 China; 6 Department of Anesthesiology, the Third Affiliated Hospital of Inner Mongolia Medical University, BaoGang Hospital, Baotou, 014010 China; 7 School of Pharmacy, Yantai University, Yantai, 264005 China

**Keywords:** Neuropeptide Y, Moderation, ApoA-I, ApoB, Cerebralspinal fluid

## Abstract

**Background:**

Apoprotein A-I (ApoA-I) and Apoprotein B (ApoB) have emerged as novel cardiovascular risk biomarkers influenced by feeding behavior. Hypothalamic appetite peptides regulate feeding behavior and impact lipoprotein levels, which effects vary in different weight states. This study explores the intricate relationship between body mass index (BMI), hypothalamic appetite peptides, and apolipoproteins with emphasis on the moderating role of body weight in the association between neuropeptide Y (NPY), ghrelin, orexin A (OXA), oxytocin in cerebrospinal fluid (CSF) and peripheral ApoA-I and ApoB.

**Methods:**

In this cross-sectional study, we included participants with a mean age of 31.77 ± 10.25 years, categorized into a normal weight (NW) (*n* = 73) and an overweight/obese (OW/OB) (*n* = 117) group based on BMI. NPY, ghrelin, OXA, and oxytocin levels in CSF were measured.

**Results:**

In the NW group, peripheral ApoA-I levels were higher, while ApoB levels were lower than in the OW/OB group (all *p* < 0.05). CSF NPY exhibited a positive correlation with peripheral ApoA-I in the NW group (*r* = 0.39, *p* = 0.001). Notably, participants with higher CSF NPY levels had higher peripheral ApoA-I levels in the NW group and lower peripheral ApoA-I levels in the OW/OB group, showing the significant moderating effect of BMI on this association (R^2^ = 0.144, β=-0.54, *p* < 0.001). The correlation between ghrelin, OXA and oxytocin in CSF and peripheral ApoB in both groups exhibited opposing trends (Ghrelin: *r* = -0.03 and *r* = 0.04; OXA: *r* = 0.23 and *r*=-0.01; Oxytocin: *r*=-0.09 and *r* = 0.04).

**Conclusion:**

This study provides hitherto undocumented evidence that BMI moderates the relationship between CSF NPY and peripheral ApoA-I levels. It also reveals the protective role of NPY in the NW population, contrasting with its risk factor role in the OW/OB population, which was associated with the at-risk for cardiovascular disease.

## Introduction

Abnormal lipoprotein metabolism represents a significant risk factor for cardiovascular disease (CVD), especially within the obese population, where CVD incidence and mortality rates are significantly increased [1]. CVD, a leading cause of global morbidity and mortality [2], affects approximately 330 million individuals, as reported in the 2019 China Cardiovascular Disease Health and Disease study [3]. As the Chinese population continues to grow and age, the prevalence of CVD continues to rise [4]. A cohort study tracking individuals for 6–19 years identified abnormal lipid metabolism as a relative risk factor for cardiovascular and all-cause mortality, particularly when high-density lipoprotein (HDL) levels fall below 1.3 mmol/L [5]. Therefore, early attention to abnormal lipoprotein profiles can help in reducing CVD risk and maintaining cardiovascular health.

Lipoproteins mainly encompass apolipoprotein A-I (ApoA-I), apolipoprotein B (ApoB), HDL, low-density lipoprotein (LDL), and very low-density lipoprotein (VLDL) [6]. LDL and HDL are the conventional cardiovascular risk biomarkers used during clinical practice [7]. Numerous clinical studies have suggested that ApoA-I and ApoB are superior predictors of cardiovascular risk compared to conventional lipid parameters like LDL and HDL [8]. ApoA-I, a primary apolipoprotein of HDL, facilitates cholesterol efflux from macrophage foam cells, initiating the reverse cholesterol transport pathway. This process involves hepatic uptake and subsequent gut excretion to prevent excessive cholesterol accumulation in macrophages [9–11]. ApoA-I also modulates the inflammatory response [12] and mitigates endothelial cell toxicity [13], making it a widely recognized cardiovascular protective factor [14]. ApoB, as the primary cholesterol transporter to the vascular artery wall, is linked to an increased risk of CVD [6, 15]. A reduction in ApoA-I or an elevation in ApoB can contribute to atherosclerosis and raise the risk of cardiovascular events [8, 16–18]. Recent research indicates that transgenic mice expressing high levels of human ApoA-I exhibit increased HDL levels, potentially offering important therapeutic insights for safeguarding against endothelial damage and reducing the incidence of CVD events [19]. While the association of ApoA-I and ApoB with CVD has been extensively documented, their interplay with feeding behavior-related hormones remains less explored [20].

Hypothalamic appetite peptides acting as appetite-stimulating neurotransmitters or neuromodulators not only regulate feeding behavior [21] but also exert a certain influence on lipid metabolism [22, 23]. These hypothalamic appetite peptides encompass neuropeptide Y (NPY), ghrelin, orexin A (OXA), and oxytocin. Central administration of NPY has been shown to elevate adiposity and plasma triglyceride (TG) levels [24–26]. Animal studies have demonstrated that NPY can enhance VLDL production in rat livers by activating the sympathetic nerves in the liver, potentially accompanied by increased levels of ApoB [27]. Additionally, another study indicated that NPY receptor activation could stimulate the expression and secretion of ApoA-I in the liver [28]. There is an increasing consensus suggesting an interaction between Ghrelin with TG-rich lipoproteins, HDL, VHDL, and certain LDL [29, 30]. A study revealed that higher serum ghrelin concentrations were associated with an elevated ApoA-I/ApoB ratio in an adult Chinese population [31]. Serum levels of OXA have shown a negative correlation with unfavorable plasma lipoproteins, specifically LDL and VLDL, in a population study related to coronary heart disease [32]. In a study involving male subjects aged 50–85, higher oxytocin levels were associated with lower levels of protective plasma lipoprotein HDL [33]. While it is evident that hypothalamic appetite peptides can impact lipoprotein metabolism, it remains essential to comprehend how body weight influences this effect.

It has been observed that appetite peptides have varying effects on lipoproteins in different weight states. Neuropeptide Y, primarily synthesized by the hypothalamus, plays a crucial role in lipid metabolism and CVD [34–36]. While NPY exhibits a negative correlation with energy expenditure in the general population [37], in the obese population, NPY levels tend to increase [37, 38] and stimulate the proliferation of peripheral white adipose tissue [39], possibly through neuron transmission or the blood-brain barrier [37]. Ghrelin, a growth hormone-releasing peptide secreted by the stomach during fasting, serves diverse biological functions [40, 41]. Serum ghrelin levels rise during fasting or in underweight patients [42] and are positively correlated with LDL and HDL levels in healthy males [43]. Interestingly, fasting CSF ghrelin levels in obese individuals are reportedly approximately 16% lower than in individuals with a normal weight [44], which may represent a protective mechanism to maintain energy balance. Ghrelin exerts direct peripheral effects on lipid metabolism, including increased white adipose tissue mass and stimulation of hepatic lipogenesis [45]. However, further research is warranted to ascertain whether different body weights influence the effect of ghrelin on peripheral lipid metabolism. OXA is produced by orexin neurons primarily located in the lateral and posterior hypothalamus, and its role in promoting appetite and lipid accumulation has been well-documented [46]. Serum OXA levels have been significantly reduced in obese individuals [47] and increased after weight loss [48]. Animal studies have revealed that OXA neurons in obese rats are sensitive to fat and are closely associated with elevated TG levels [49]. Oxytocin and its receptors have been implicated in regulating energy metabolism and food intake. Several studies have shown that oxytocin levels are positively correlated with BMI [33, 50]. Conversely, a study revealed that in obese patients, oxytocin levels were decreased, accompanied by an increase in TG and LDL levels. Oxytocin exhibited a negative correlation with LDL and TG [51]. Different BMI statuses result in varying oxytocin expressions and lipid metabolism, highlighting the influence of body weight on the relationship between appetite peptides and lipid metabolism.

Although ApoA-I and ApoB are currently considered better risk markers of CVD than lipoproteins, current research predominantly focuses on the impact of peripheral appetite peptides on lipoproteins despite appetite regulation primarily originating in the central nervous system [52]. Therefore, this study aims to investigate the relationship between four appetite peptides in CSF and ApoA-I and ApoB in obese individuals and explore how body weight influences appetite peptides and lipid metabolism. These findings may provide valuable insights for preventing and treating dyslipidemia and CVD.

## Materials and methods

### Participants

In this study, we recruited 190 Chinese men between September 2014 and January 2016. Participants were patients who had planned to undergo anterior cruciate ligament reconstruction surgery. According to the obesity diagnostic criteria outlined in the “Guidelines for the Prevention and Control of Overweight and Obesity in Chinese Adults”, the participants were divided into two groups: the normal weight group (NW), consisting of 117 individuals (with a BMI ranging from 18.5 kg/m² to less than 24 kg/m²), and the overweight/obese group (OW/OB), comprising 73 individuals (with a BMI of 24 kg/m² or higher). Besides sociodemographic data such as age, years of education, and smoking status, we also collected information on substance abuse and dependence through self-reporting, which was subsequently verified by the subjects’ next of kin and family members.

The exclusion criteria for this study encompassed the following: (1) a family history of mental or nervous system diseases or central nervous system disorders, as assessed through the international neuropsychiatric interview; (2) systemic diseases identified through medical history and admission diagnosis; and (3) individuals taking lipid-lowering medications. None of the participants had a history of drug dependence or abuse, including cigarettes, which was confirmed by their next of kin.

This study received approval from the Institutional Review Committee of Inner Mongolia Medical University (approval number: YKD2015003) and adhered to the principles of the Helsinki Declaration. To ensure that both the subjects and their guardians comprehended the study’s content, an anesthesiologist explained the research plan. We obtained written informed consent from adult participants and the guardians of minors. No financial compensation was provided to the subjects in this study.

### Assessments, biological sample collection, and laboratory tests

Upon admission, the height and weight of the subjects were measured with an accuracy of 0.5 cm and 0.1 kg, respectively, to calculate the body mass index (BMI) using the formula BMI (kg/m^2^) = weight (kg)/ height (m)^2^. All subjects were required to fast for at least 8 h overnight, after which peripheral venous blood was drawn from the elbow in the morning on an empty stomach for various analyses, including blood routine, liver function, renal function, and blood lipid levels. These analyses included alanine aminotransferase (ALT), aspartate aminotransferase (AST), γ-glutamyltransferase (GGT), total cholesterol (CHO), TG, HDL, LDL, ApoA-I and ApoB. The measurements were conducted using a biochemical automatic analyzer (HITACH 7600, Hitachi, Tokyo, Japan).

For patients undergoing anterior cruciate ligament reconstruction in China, lumbar puncture is a routine part of the preoperative procedure. In the morning before surgery, an anesthesiologist performed lumbar puncture and collected 5 ml of CSF sample and 3 ml of peripheral blood samples. Each CSF and plasma sample was transferred to 0.5 ml test tubes and frozen at -80 °C.

The quantification of OXA levels in CSF was carried out using an enzyme-linked immunosorbent assay kit (Cloud Clone Corp., Houston, TX, USA). Additionally, NPY and oxytocin levels were assessed using radioimmunoassay kits (Phoenix Pharmaceuticals, Inc., Burlingame, CA, USA), and Ghrelin levels were measured using a radioimmunoassay kit (DIAsource ImmunoAssays S.A., Louvain-la-Neuve, Belgium). Laboratory technicians conducting these analyses were blinded to clinical data.

### Statistical analysis

The Shapiro-Wilk and Levene tests were used to assess the distribution normality and variance homogeneity for continuous variables, respectively. Continuous variables with homogeneity of variance were compared using unpaired t-test, while the variables without homogeneity of variance, such as TG, GGT, and NPY, were used the Mann-Whitney test to compare differences between groups. Categorical variables were assessed using the chi-square test. Descriptive statistics, such as means and standard deviations (SD), were used for continuous variables, whereas frequencies and percentages were employed for categorical variables. Correlation analysis was conducted using partial correlation, adjusting for smoking status. Hierarchical regression was used to analyze the moderating effect, with adjustments for smoking status as covariates, and bonferroni correction for multiple comparisons was applied. A simple slope test was performed to assess the moderating effect in cases where the moderating effect was statistically significant. All statistical analyses were conducted using IBM SPSS, version 27.0 (IBM, Armonk, N.Y., USA), and the chart was generated using the corrplot function in the R programming language 4.3.1. All tests were two-tailed, with a significance threshold set at *p* < 0.05.

## Results

### Demographic and clinical characteristics

In comparison to the OW/OB group, the NW group had a lower rate of smoking and higher levels of HDL (1.34 ± 0.30 vs. 1.21 ± 0.32), ApoA-I (1.55 ± 0.59 vs. 1.43 ± 0.19), and CSF Ghrelin (1501.65 ± 194.90 vs. 1455.30 ± 229.65). Conversely, the NW group had lower levels of LDL (2.45 ± 0.61 vs. 2.81 ± 0.75), ALT (25.72 ± 22.27 vs. 33.51 ± 21.93), CHO (4.47 ± 0.92 vs. 4.84 ± 0.96), TG (1.48 ± 1.11 vs. 2.05 ± 0.91), GGT (30.28 ± 26.77 vs. 48.25 ± 36.28), and ApoB (0.89 ± 0.23 vs. 1.03 ± 0.24) (all *p* < 0.05, Table 1). There were no significant differences in age, years of education, NPY, OXA, and oxytocin levels in CSF between the two groups (all *p* > 0.05, Table 1).

**Table 1:** Demographic and clinical characteristics of groups

Variables	NW(*n* = 73)	OW/OB(*n* = 117)	F/t/z	*p*
Smoke status, n(%)				
Non-smokers	48(65.8)	55(47.0)	6.36	0.012*
Active-smokers	25(34.2)	62(53.0)		
Age(years)	29.89 ± 11.14	31.31 ± 9.37	-0.87	0.388
Education (years)	12.98 ± 2.77	12.73 ± 2.78	0.81	0.421
HDL (mmol/L)	1.34 ± 0.30	1.21 ± 0.32	3.29	0.001*
LDL (mmol/L)	2.45 ± 0.61	2.81 ± 0.75	-3.74	< 0.001*
ALT (U/L)	25.72 ± 22.27	33.51 ± 21.93	-2.50	0.013*
CHO (mmol/L)	4.47 ± 0.92	4.84 ± 0.96	-3.12	0.002*
TG (mmol/L)	1.48 ± 1.11	2.05 ± 0.91	-5.22	< 0.001*
GGT(U/L)	30.28 ± 26.77	48.25 ± 36.28	-5.28	< 0.001*
AST(U/L)	19.74 ± 9.01	22.17 ± 8.73	-1.84	0.068
ApoA-I(g/L)	1.55 ± 0.59	1.43 ± 0.19	2.05	0.042*
ApoB(g/L)	0.89 ± 0.23	1.03 ± 0.24	-4.42	< 0.001*
CSF NPY(pg/ml)	170.95 ± 28.06	173.92 ± 31.64	-0.49	0.623
CSF Ghrelin (pg/ml)	1501.65 ± 194.90	1455.30 ± 229.65	2.12	0.035*
CSF OXA (ng/ml)	183.74 ± 31.88	176.82 ± 39.24	0.99	0.321
CSF Oxytocin (pg/ml)	45.03 ± 8.08	44.94 ± 8.50	0.07	0.942

*Abbreviations* HDL, high-density lipoprotein; LDL, low-density lipoprotein; ALT, alanine aminotransferase; CHO, cholesterol; TG, triglyceride; GGT, gamma-glutamyl transferase; AST, aspartate aminotransferase; ApoA-I, Apolipoprotein A-I; ApoB, Apolipoprotein B; CSF, cerebral spinal fluid; NPY, neuropeptide Y; OXA, Orexin A; NW, normal weight group; OW/OB, overweight/obese group

*Note* All data were reported by mean ± SD and using unpaired t-test, in addition to smoking status using Chi-square test, and TG, GGT, CSF NPY using Mann Whitney test, *p < 0.05

### Correlation analysis

**Fig. 1:**
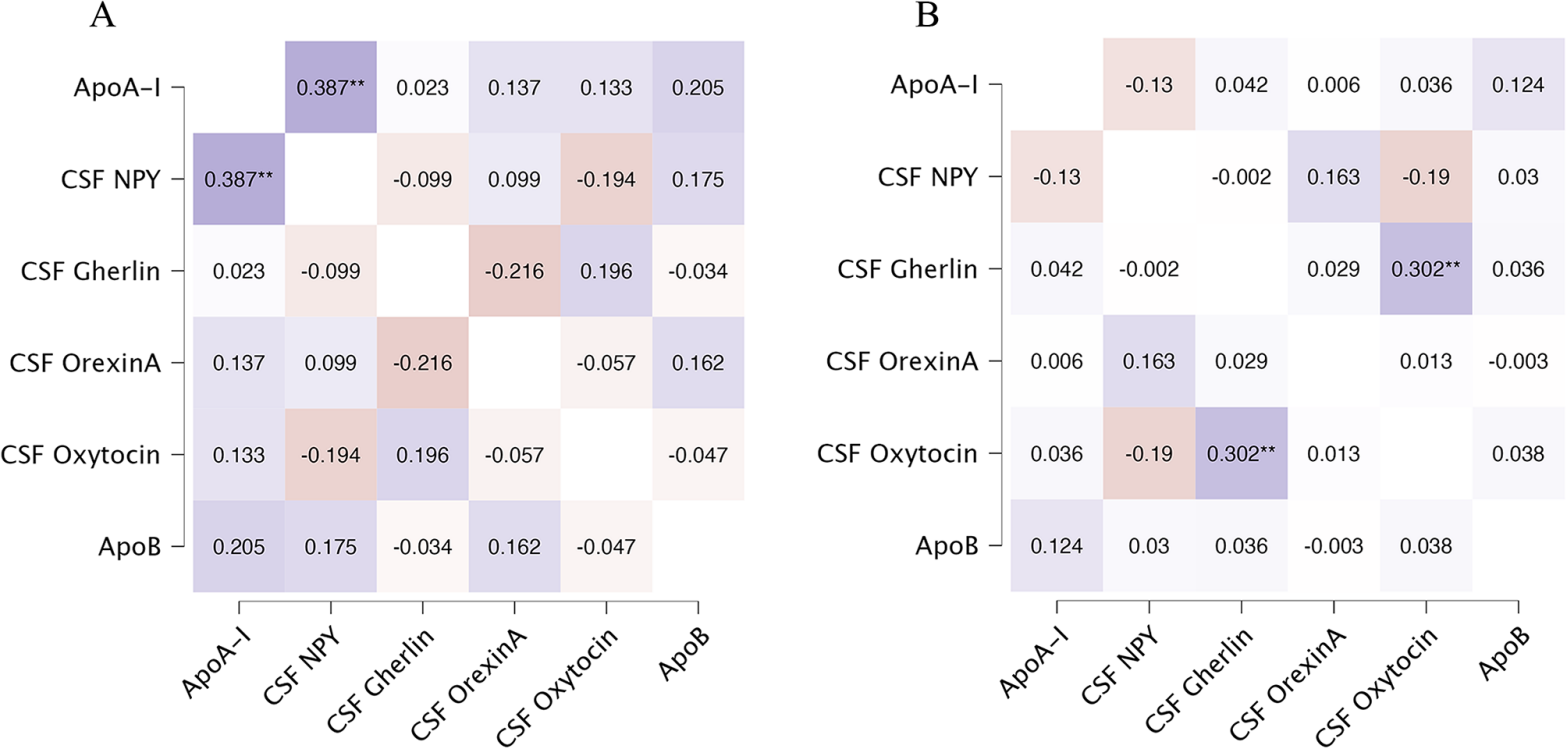
Correlation of CSF appetite peptide with peripheral ApoA-I and ApoB between NW group and OW/OB group. Note: Adjusted for smoke status. *p < 0.05, **p < 0.01. **(A)** The correlation between CSF appetite peptide and peripheral ApoA-I and ApoB in the NW group. **(B)** The correlation between general demographic variables, CSF appetite peptide, and peripheral ApoA-I and ApoB in the OW/OB group

To investigate the relationship between CSF appetite peptides and peripheral ApoA-I and ApoB in the two groups, we conducted a partial correlation analysis, adjusting for smoking status [53] (Table 2; Fig. 1). In the NW group, a positive correlation was observed between CSF NPY and peripheral ApoA-I (*r* = 0.39, *p* = 0.001), while no correlation was found in the OW/OB group (*p* > 0.05). Notably, the correlation between CSF NPY and peripheral ApoA-I in the NW and OW/OB groups exhibited opposite directions (NPY: *r* = 0.39 and *r*=-0.13). Neither group had a significant correlation between CSF NPY and peripheral ApoB (*p* > 0.05). Furthermore, there was no correlation between CSF ghrelin, OXA, oxytocin, and peripheral ApoA-I and ApoB (all *p* > 0.05). However, the correlation between ghrelin, OXA and oxytocin levels in CSF and peripheral ApoB in the NW and OW/OB groups exhibited opposite trends (Ghrelin: *r* = -0.03 and *r* = 0.04; OXA: *r* = 0.23 and *r*=-0.01; Oxytocin: *r*=-0.09 and *r* = 0.04, Table 2; Fig. 1). These findings suggest that different weight groups may influence the relationship between NPY, ghrelin, OXA, and oxytocin in CSF and peripheral ApoA-I and ApoB. To confirm these hypotheses, we constructed a moderated model to assess the role of BMI in the relationship between the four appetite peptides in CSF and peripheral ApoA-I and ApoB, respectively.

**Table 2:** Partial correlation analysis between CSF appetite hormone and peripheral ApoA-I and ApoB

Groups	Variables	ApoA-I		ApoB	
		r	*p*	r	*p*
NW	CSF NPY(pg/ml)	0.39	0.001*	0.18	0.153
	CSF ghrelin	0.02	0.851	-0.03	0.780
	CSF OXA	0.14	0.277	0.16	0.196
	CSF Oxytocin	0.13	0.336	-0.05	0.736
OW/OB	CSF NPY(pg/ml)	-0.13	0.188	0.03	0.761
	CSF ghrelin	0.04	0.669	0.04	0.718
	CSF OXA	0.01	0.955	-0.00	0.978
	CSF Oxytocin	0.04	0.756	0.04	0.746

*Abbreviations* ApoA-I, Apolipoprotein A-I; ApoB, Apolipoprotein B; CSF, cerebral spinal fluid; NPY, neuropeptide Y; OXA, Orexin A; NW, normal weight group; OW/OB, overweight/obese group

*Note* Partial correlation was used for the calculation of associations between variables. *p* < 0.05 was considered significant. *p < 0.05

Adjusted for smoke status

### Moderation analysis for CSF appetite peptides and peripheral ApoA-I and ApoB

We next performed hierarchical multiple regression analysis on the peripheral ApoA-I, and standardized all variables. In Model 1, where peripheral ApoA-I served as the dependent variable, we included smoking status, BMI groups, and CSF NPY. Model 2 introduced the interaction term BMI groups×NPY.

The results from Model 2 indicated that, after controlling for smoking status, BMI groups exhibited a negative correlation with peripheral ApoA-I (β=-0.16, *p* < 0.001), while CSF NPY displayed a positive correlation with peripheral ApoA-I (β = 0.61, *p* < 0.001). Importantly, the BMI groups×NPY interaction was negatively correlated with peripheral ApoA-I (β=-0.54, *p* < 0.001) (Table 3).

**Table 3:** Hierarchical multiple regression of peripheral ApoA-I

Model	Variables	ApoA-I				
		B	SE B	β	t	*p*
1	Smoke status	-0.07	0.06	-0.09	-1.15	0.251
	BMI groups	-0.11	0.06	-0.15	-1.91	0.058
	CSF NPY(pg/ml)	0.06	0.03	0.16	2.02	0.045
2	Smoke status	-0.08	0.06	-0.11	-1.40	0.165
	BMI groups	-0.12	0.06	-0.16	-2.16	0.032
	CSF NPY(pg/ml)	0.23	0.05	0.61	4.76	< 0.001*
	BMI groups × CSF NPY	-0.25	0.06	-0.54	-4.33	< 0.001*

*Abbreviations* B, non-standardized coefficient; SE, standard error; β, standardized coefficient beta; ApoA-I, Apolipoprotein A-I; CSF, cerebral spinal fluid; NPY, neuropeptide Y

*Note* Hierarchical multiple regression was used to analyze ApoA-I. All data have been standardized. Smoke status, BMI groups, and CSF NPY were entered in Step 1; BMI groups×CSF NPY were entered in Step 2. *Significant after Bonferroni correction for multiple comparisons

**Fig. 2:**
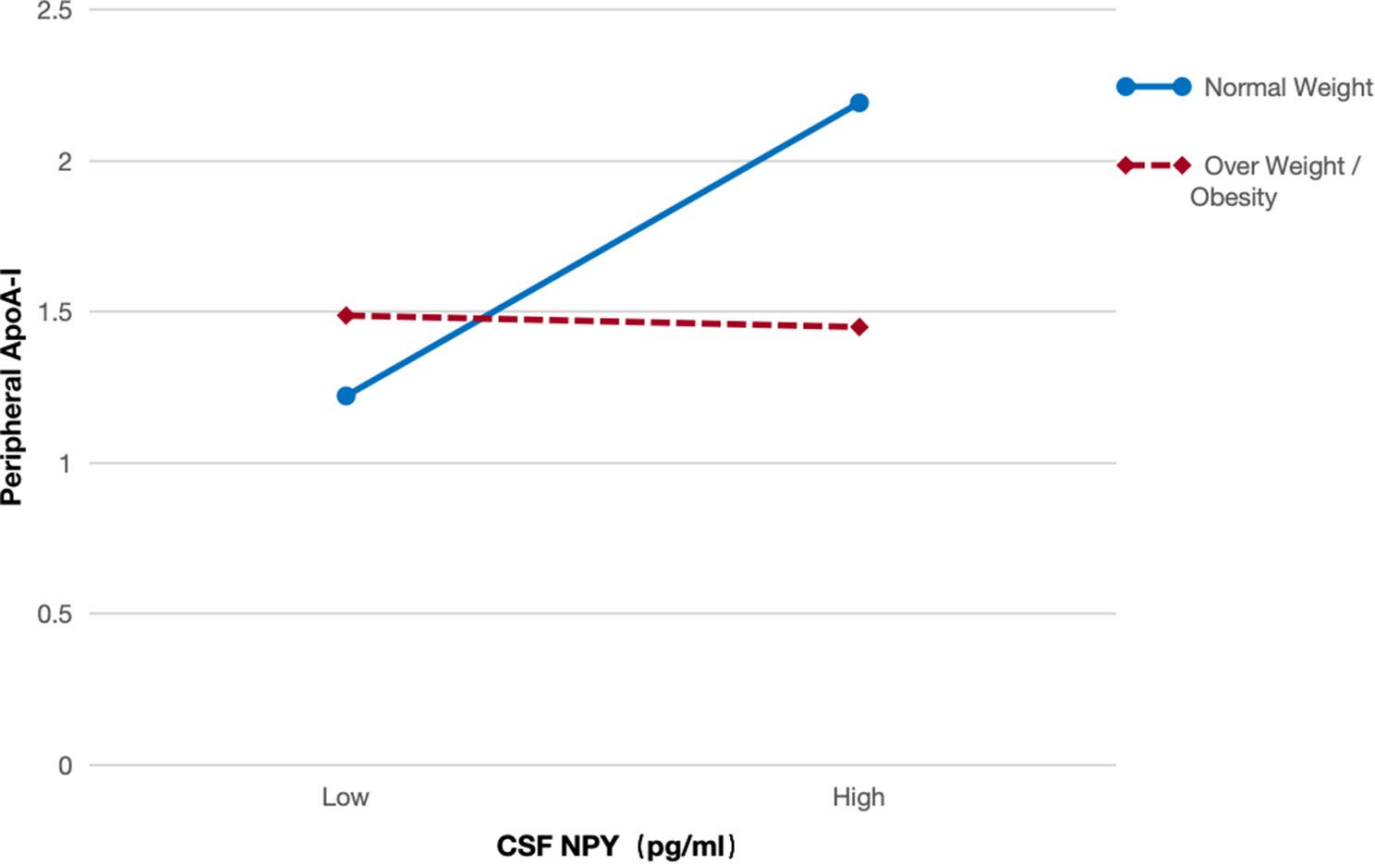
Simple slope test of the interaction between BMIG and CSF NPY on peripheral ApoA-I

To further explore the moderating effect of BMI, we conducted a simple slope analysis. In the NW group, individuals with higher levels of CSF NPY exhibited a significantly higher level of peripheral ApoA-I. In contrast, in the OW/OB group, individuals with higher levels of CSF NPY displayed a significantly lower level of peripheral ApoA-I (Fig. 2).

We employed the same hierarchical multiple regression analysis method to examine the influence of BMI on ghrelin, OXA, and oxytocin in CSF and their relationship with peripheral ApoA-I. The results revealed that BMI did not moderate the relationship between ghrelin, OXA, and oxytocin in CSF and peripheral ApoA-I (all *p >* 0.05). Additionally, analysis of the impact of BMI on the four appetite peptides in CSF and their relationship with peripheral ApoB showed that BMI did not regulate these relationships (all *p >* 0.05).

## Discussion

This study marks the first attempt to explore the moderating effect of body weight on appetite peptides NPY, ghrelin, OXA, and oxytocin in CSF, and peripheral ApoA-I and ApoB in males. While many studies have employed ApoB/ApoA-I as a predictor of cardiovascular risk [17], it remains unclear whether the correlation between appetite peptides and ApoB/ApoA-I hinges on the presence of ApoA-I or ApoB alone or their combination through the ApoB/ApoA-I ratio. Therefore, we analyzed the relationships between appetite peptides and ApoA-I and ApoB, as well as the moderating role of body weight in these relationships. Our primary findings revealed that different BMI groups moderated the relationship between CSF NPY and peripheral ApoA-I. In individuals with normal weight, high CSF NPY levels were associated with high peripheral ApoA-I levels, indicating that NPY is a protective factor for peripheral ApoA-I. In contrast, among OW/OB individuals, high CSF NPY levels were linked to low peripheral ApoA-I levels, making NPY a risk factor.

It is widely acknowledged that NPY is synthesized within the arcuate nucleus of the hypothalamus and transported to the paraventricular nucleus via axons for secretion, participating in the regulation of food intake [54]. Research has shown that intracerebroventricular administration of NPY in normal-weight rats induces hyperphagia, weight gain, and increased fat mass accumulation [55]. NPY acts as a communication bridge between the hypothalamus and adipose tissue, with circulating NPY levels closely related to the central brain source of NPY. It can be integrated into the metabolic response of both the central and peripheral nervous systems [56]. The central nervous system (CNS) NPY potentially regulates peripheral lipoproteins through the hypothalamus-sympathetic nervous system-adipose tissue innervation pathway and within adipose tissue [39]. Animal studies have shown that the activation of NPY receptors in normal-weight rats can promote the synthesis and secretion of ApoA-I in hepatocytes through the extracellular signal-regulated kinase 1/2 and protein kinase A signal transduction pathways [28]. Although there is no direct evidence of the impact of intracerebroventricular NPY injection on ApoA-I, Y5 receptors, primarily expressed in the CNS [57], act as a peripheral signal of energy availability [58]. CSF NPY can directly access circulating hormones due to a semi-permeable blood-brain barrier [59]. Therefore, it is highly conceivable that CNS NPY also promotes the secretion of ApoA-I in the liver through these channels. ApoA-I is the principal apolipoprotein of HDL and plays a crucial role in cholesterol metabolism. It removes cholesterol from tissues, transports cholesterol back to the liver from arterial walls, and facilitates its excretion through bile [60, 61]. In the NW population, increased CSF NPY levels promoted food intake and fat accumulation and enhanced cholesterol efflux, modulating free cholesterol and cholesterol esters in cells to maintain liver lipid metabolism balance. This, in turn, prevented lipid accumulation and reduces the formation of foam cells [62], potentially preventing atherosclerosis [63]. Hence, in this study, an increase in NPY levels was associated with higher ApoA-I concentrations, a potentially protective mechanism to maintain lipid balance in the liver [64, 65].

Animal experiments revealed chronically elevated hypothalamic NPY activity in genetically obese ob/ob mice [66]. The expression of NPY mRNA increased in the hypothalamus of rats that are made obese by an early diet [67], particularly in cases of chronic obesity where NPY was highly expressed in the dorsal hypothalamic nucleus [68, 69]. CSF NPY levels have been reported to significantly increase with body weight [37, 38]. While NPY is predominantly secreted by CNS neurons, there are lower concentrations in the peripheral system [39]. Thus, the effect of CNS NPY on peripheral adipose tissue may be achieved indirectly through bidirectional neuronal and hormonal communication and possibly through direct circulation across the blood-brain barrier [39]. NPY is secreted by sympathetic neurons to regulate inflammation [70]. In obese tissues, NPY binds to macrophage Y1 receptors, increasing cholesterol uptake and intracellular cholesterol content. It significantly inhibits the efflux of extracellular cholesterol receptors ApoA1 and HDL in macrophages [71], reducing peripheral ApoA-I levels. This process is accomplished by decreasing the expression of ATP-binding cassette transporter A1, ATP-binding cassette G1, and scavenger receptor B1 in obese tissues [72–74]. These findings suggest that ApoA-I levels are decreased with increasing CSF NPY levels among obese individuals.

Another noteworthy discovery in this study is the absence of an interaction between CSF NPY and peripheral ApoB and the interaction between CSF ghrelin, OXA, and oxytocin and peripheral ApoA-I and ApoB. In lean rats, intracerebroventricular administration of NPY into the third ventricle was found to stimulate sympathetic innervation of the liver and enhance VLDL-TG secretion through the CNS NPY Y1 receptor [24, 75]. However, in mice, acute central administration of NPY did not affect hepatic VLDL production [27]. This difference may be attributed to variations in basal hepatic VLDL-TG production rates between rats and mice [24, 76], indicating that species-specific factors influence hepatic VLDL metabolism. The interspecies differences may influence ApoB, a critical apolipoprotein in VLDL [77]. Genetic association studies in humans have reported conflicting results regarding the role of NPY gene and receptor polymorphisms in serum TG metabolism [78, 79], further underscoring the impact of species differences. This variability might explain why no correlation between CSF NPY and ApoB was observed in this study.

Ghrelin is the only appetite peptide hormone produced by the stomach [80], which promotes the uptake and synthesis of lipids in the fat and liver, and inhibits lipolysis [81]. Some studies have reported that peripheral ghrelin affects TC, LDL, HDL, and more [29, 30, 82]. In the human bloodstream, ghrelin circulates in two forms: octanoylated ghrelin and deacylated ghrelin, which interact with ApoB-containing and ApoA-I-containing lipoproteins, respectively [30]. However, our study found that there was no correlation between central ghrelin and peripheral ApoA-I and ApoB. On the one hand, circulating ghrelin crosses the blood-brain barrier and indirectly promotes appetite by binding to growth hormone secretagogue receptor 1a (GHS-R1a), which has no direct effect on peripheral lipoprotein. On the other hand, although studies have confirmed that central ghrelin can also directly regulate adipocyte metabolism [41], its role is reflected in increasing the expression of fat storage–promoting enzymes in white adipocytes and decreasing the expression of the thermogenesis-related mitochondrial uncoupling proteins 1 and 3 in brown adipocytes, and no direct effect on lipoprotein has been reported [41]. Thus, further investigation is required to better understand the impact of CSF ghrelin on peripheral ApoA-I and ApoB.

The central OXA is able to cross the blood-brain barrier and promote lipogenesis and inhibit lipolysis in peripheral tissues [83, 84]. A clinical study revealed that individuals in the OXA increase group experienced a greater decrease in serum ApoB after bariatric surgery compared to the OXA decreased group. These results indicate a positive association between increasing orexin levels and ApoB improvement [85]. However, these conflicting findings may be attributable to variations in the action of OXA receptors, emphasizing the need for further research to elucidate the specific mechanisms. Our study found no association between OXA and apolipoproteins, which may also be influenced by gender. Notably, endogenous androgens have been found to reduce the activation of OXA neurons in males [86]. This factor might contribute to our inability to obtain positive results. Furthermore, the influence of CNS OXA on energy metabolism primarily centers on the thermogenesis and energy consumption of brown adipose tissue (BAT). Therefore, further research is needed to determine whether these effects align with lipid accumulation in white adipose tissue (WAT).

It was reported that the oxytocin receptor increases energy expenditure by stimulating BAT thermogenesis and promoting the browning of WAT [87]. Nevertheless, this study did not reveal a relationship between oxytocin and peripheral apolipoproteins, possibly because oxytocin promotes the browning of WAT rather than its formation. Therefore, it may not affect triglyceride accumulation or apolipoprotein levels.

Several limitations should be noted in this study. Firstly, the study participants were individuals with anterior cruciate ligament injuries, and the preoperative stress response in these participants might have influenced CSF appetite peptide levels [88]. Nonetheless, central CSF samples reflect more accurately human neuroendocrine metabolism than blood samples. Secondly, the study included only male participants. To generalize the results, further studies with female participants should be conducted. Lastly, the study did not include a low-weight group (BMI < 18.5 kg/m^2^). It is known that the secretion of appetite peptides varies in individuals with low body weight [44, 89, 90], which may have different effects on peripheral ApoA-I and ApoB. Further research is warranted to explore this specific scenario.

## Conclusion

This study provides preliminary evidence of the moderating role of BMI in the relationship between CSF NPY and peripheral ApoA-I levels. Our findings reveal that NPY plays a protective role in individuals within the normal weight range while acting as a risk factor for those who are overweight or obese. This distinction is crucial, given that it is associated with risk for cardiovascular disease.

## Data Availability

The data that support the findings of this study are available from the corresponding authors. Requests to access these datasets should be directed to Fan Wang.

## Abbreviations

ApoA-I: Apolipoprotein A-I
ApoB: Apolipoprotein B
BMI: Body mass index
NPY: Neuropeptide Y
OXA: Orexin A
CSF: Cerebralspinal fluid
NW: Normal weight
OW/OB: Over weight/obese
CVD: Cardiovascular disease
HDL: High-density lipoprotein
LDL: Low-density lipoprotein
VLDL: Very low-density lipoprotein
TG: Triglyceride
VHDL: Very high-density lipoprotein
ALT: Alanine aminotransferase
AST: Aspartate aminotransferase
GGT: Gamma-glutamyl transferase
CHO: Cholesterol
ANOVA: One-way analysis of variance
SD: Standard deviation
CNS: Central nervous system
BAT: Brown adipose tissue
WAT: White adipose tissue
